# Editorial: Enviromics in Plant Breeding

**DOI:** 10.3389/fpls.2022.935380

**Published:** 2022-06-30

**Authors:** Rafael Tassinari Resende, Karine Chenu, Soren K. Rasmussen, Alexandre Bryan Heinemann, Roberto Fritsche-Neto

**Affiliations:** ^1^Universidade Federal de Goiás, Plant Breeding Sector, Goiânia, Brazil; ^2^Queensland Alliance for Agriculture and Food Innovation, University of Queensland, St Lucia, QLD, Australia; ^3^Section for Plant Biochemistry, Department of Plant and Environmental Sciences, Faculty of Natural and Life Sciences, University of Copenhagen, Frederiksberg, Denmark; ^4^Embrapa Arroz e Feijão, Santo Antônio de Goiás, Brazil; ^5^International Rice Research Institute, Los Baños, Philippines

**Keywords:** Ambientómica, Ambientômica, Umweltomik, omics, precision breeding, geoinformatics

## Key Message

Enviromics is a process dealing with environmental characterization by envirotyping for micro- or macro-environments. In plant science, efforts have focused on interpretating plant responses to environmental cues and on understanding the interactions of genotypes with environments.

Agronomy and plant breeding aim to improve crop productivity and sustainability. That said, cultivars should ideally be developed for the *Target Population of Environments* (TPE) and grown in environments that allow their best performance (Chenu, 2015). However, spatial and temporal heterogeneity of environmental conditions, genotype-by-environment (*G*×*E*) interactions, and/or genotype-by-environment-by-management (*G*×*E*×*M*) interactions can bound breeding progress. It is well-known that various environmental components cause phenotypic variations to plant growth and development. However, knowing the fullness of environmental effects that contribute to a phenotype expression (and all their interactions) is a major challenge, since quantitative traits can be influenced by a complex network of environmental cues. To compound this dilemma, phenotypic expression of traits, including yield, is expected to evolve with climatic changes associated with global warming. This is currently a key topic for improving productivity, and it makes perfect sense when we see how genes are expressed in response to spatial environmental variations.

*Envirotyping* and *enviromics* have been used in micro- and macro-environments to decipher the G×E interactions at the field and TPE levels (Chenu et al., 2011; Cooper et al., 2014; Resende et al., 2020). Here, each experimental trial is not a target, merely a sample reinforcement for what really matters: the whole area of genotypic recommendation. Approaches range from coarse to fine characterization of the environments (Cooper et al.), both in terms of spatial consideration (subfield to region) and the environmental variables considered. For instance, characterization has been done using basic climatic variables or complex crop-level factors that account for feedback between the crop and its environments (e.g., drought index computed with crop modeling to account for the feedback of plant growth on soil water depletion). Those variables can also be defined as a “coarse” averaged value over the season or a “fine” dynamic over the crop cycle (Chenu, 2015). New technologies have encouraged the development of enviromics based on a wide range of predictors from complex ecophysiology that can be used in the modeling of *G*×*E* or *G*×*E*×*M* interactions.

In this Research Topic*, “Enviromics in Plant Breeding”*, a case study of maize yield improvement in multiple-environment trials is discussed for both *G*×*E* and *G*×*E*×*M* modeling (Cooper et al.). In addition, a detailed study of enviromic-aided genomic prediction is presented by Costa-Neto et al., who propose a new algorithm to target yield stability.

**Figure 1:**
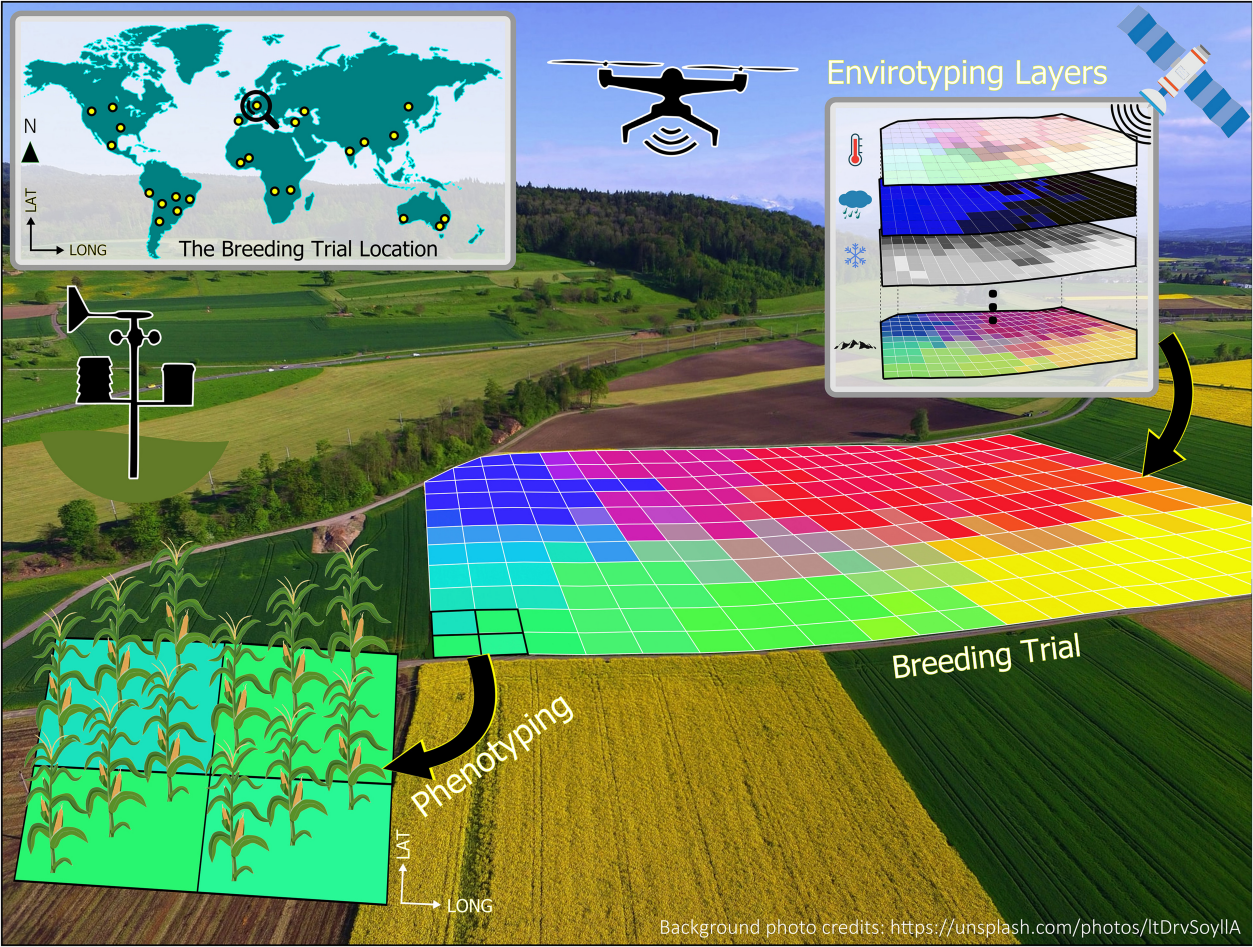
Geoprocessing environment allocated to a breeding trial, containing numerous pixels (i.e., the colored gridded cells) with multiple environmental information (e.g., climate, atmospheric, soil, and landscape) from diverse sources such as weather stations, orbital satellites, and drones. Each pixel may contain information obtained by envirotyping, phenotyping, and genotyping.

To illustrate the very basic concept of enviromics, Figure 1 presents the enviromics approach of a breeding trial, where each single-colored square from the 2D grid is understood as a *pixel*, and its envirotyped information can be related to measured phenotypes to better understand G×E interactions. The environmental similarity of plots within a breeding trial can be locally assessed with spatial models to better understand the behavior of genotypes (Cursi et al.). Crop models can also be applied for environmental characterization to define the main abiotic stress patterns as illustrated for water and heat stress across the American sorghum belt (Carcedo et al.). In this Research Topic, Oteng-Frimpong et al. also used factorial regression to analyze the major environmental covariables underlying G×E interactions in groundnuts in Ghana. Moving outside planet Earth, Mohanta et al. proposed using plants grown in outer space, which have been exposed to cosmic radiation and microgravity, to produce genotypes capable of surviving extreme environmental conditions.

We can easily perceive the importance of enviromics analyses, a modern “omics” capable of providing information resulting from the interactions of plant development subjected to complex environmental factors. Analogously to the bioinformatics development, in modern studies of *precision breeding*, the “*E*” component of the *G* × *E* interaction can best be described by “geoinformatics” procedures, casting multiple enviromic markers supporting wide-scale environmental breeding prediction (Resende et al., 2020). Knowing how to deal with high-throughput amounts of envirotyping data (Xu, 2016; Costa-Neto et al., 2021), considering *within* and *across* trials scale as well as the TPE, becomes a fundamental attribute in the prediction for representative environments that may never have been experimentally tested.

## Concluding Remarks

Enviromics can benefit from several processes in the *precision breeding* framework, to improve genetic prediction for the benefit of crop improvement. Recent studies have shown promising results from combining it with other omics and spatial analyses. Yet some challenges remain in respect to its broader deployment and the need to deal with increasingly large volumes of data.

## Author Contributions

RTR drafted the first version of the manuscript. All authors subsequently contributed to it by editing and formatting the final version.

## Conflict of Interest

The authors declare that the research was conducted in the absence of any commercial or financial relationships that could be construed as a potential conflict of interest.

## Publisher's Note

All claims expressed in this article are solely those of the authors and do not necessarily represent those of their affiliated organizations, or those of the publisher, the editors and the reviewers. Any product that may be evaluated in this article, or claim that may be made by its manufacturer, is not guaranteed or endorsed by the publisher.
